# Vive La Différence: An Interview with Catherine Dulac

**DOI:** 10.1371/journal.pgen.1002140

**Published:** 2011-06-23

**Authors:** Jane Gitschier

**Affiliations:** Department of Medicine and Pediatrics, University of California San Francisco, San Francisco, California, United States of America

One of the joyful aspects of a life in science is listening to the rare seminar that simply knocks your socks off. Two such memorable moments for me came in the form of job seminars by a pair of Richard Axel's post-docs: first, Linda Buck, who described olfactory receptors and later shared the Nobel Prize with Axel for this work, and then, Catherine Dulac, who identified pheromone receptors in the vomeronasal organ (VNO) in mice. These two sensory systems elicit very different responses: while the main olfactory system senses hundreds of thousands of different odorants, by binding volatile compounds to olfactory receptors within the main olfactory epithelium (MOE) and projecting them onto the olfactory cortex, the VNO detects a limited repertoire of species-specific pheromones that trigger sex-specific behaviors without any cortical output whatsoever.

In January, as I was heading to Cambridge where Dulac (Image 1) is now Chair of Molecular and Cellular Biology at Harvard, I was reminded of my captivation with the VNO and refreshed my memory of her work. Over the past decade she has delineated some of the molecular components of its signal transduction system, including a VNO-specific ion channel called TRPC2. Interestingly, incapacitation of the VNO, by knock-out of *Trpc2*, renders male mice unable to discriminate sexes and relaxes male aggression, while unmasking the male courtship behaviors in females, including even ultrasonic vocalization. My interest in speaking with Dulac was doubly piqued by her recent beautiful studies on genetic imprinting in the mouse brain. As both pheromones and sexual dimorphisms in the brain are topics that transcend scientific inquiry by impacting popular culture and medicine, I was keen to learn more.

**Figure:**
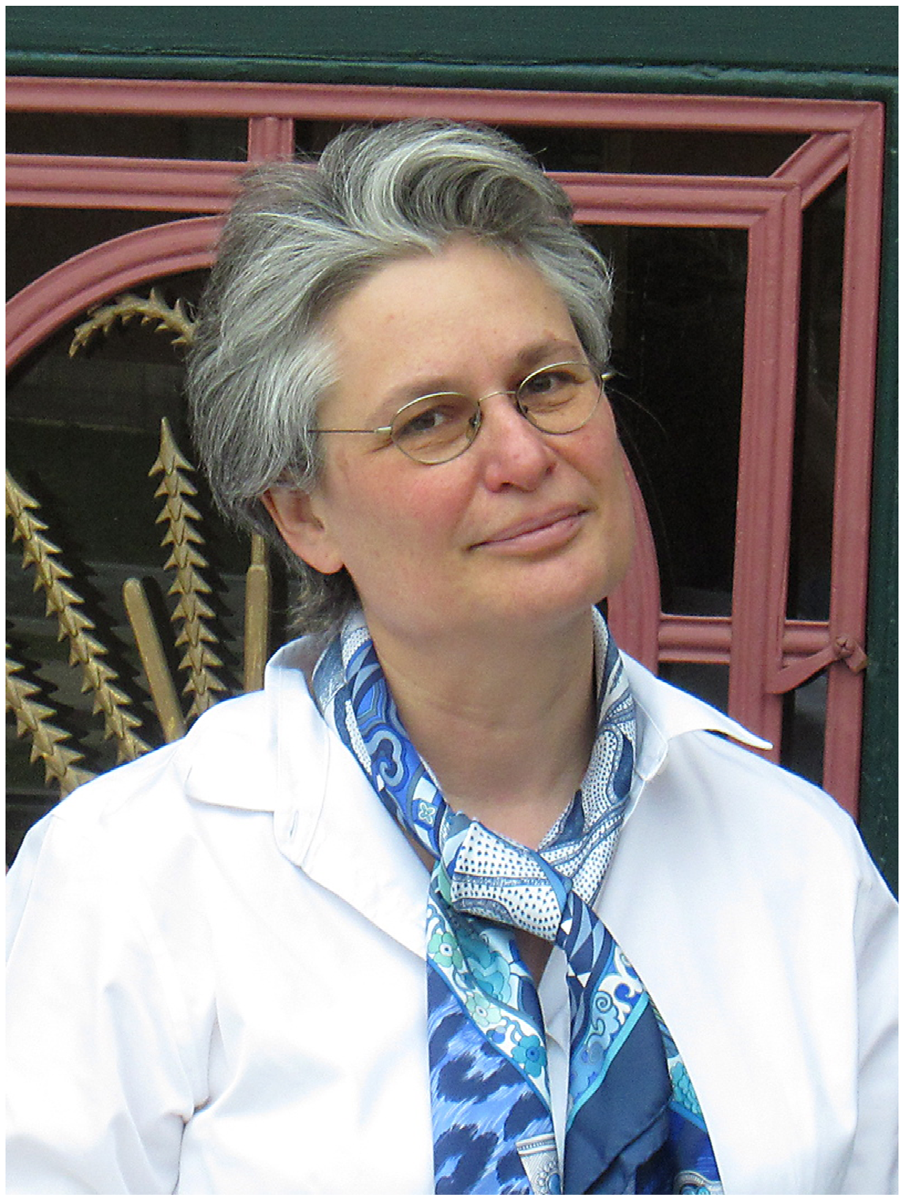
Catherine Dulac. Image courtesy of Department of Molecular and Cellular Biology, Harvard University.

Luckily, our schedules meshed and I was able to stop by her office in the massive brick Bio Labs building on Divinity Avenue. I crunched and skated my way through its courtyard, piled high with snow as Cambridge headed for its near-record accumulation of 60″ in January alone. I gave the old bronze rhinoceros at the doorway a few affectionate pats, dusting off that afternoon's snowflakes.

**Gitschier:** Let's talk about your childhood in France and how you became curious about science.

**Dulac:** I grew up in the south of France on the Mediterranean seashore where I loved to eat oysters—I'm an oyster fanatic. I was living in one of the two biggest oyster-producing areas in France.

**Gitschier:** Where specifically?

**Dulac:** Montpellier. It has one of the two oldest medical schools in Europe. It's a big scholarly place, although it is a small city. I did all my education there through high school, and then for my higher education I went to Paris.

And how did I come to be a scientist? Well, I think there are two reasons. One, I've always been fascinated by research. As a kid, I read books about history and in particular about pre-history and digging bones of old creatures. That seemed to be what I wanted to do for all my life.

**Gitschier:** What age are we talking about?

**Dulac:** As soon as I was able to read, basically. I loved imagining how people were in the past and trying to ask questions about what they were thinking and doing and making. Just the idea of being confronted with a black box and being able to ask a question and find the tools to answer the question, whether that was related to an animal or life or history.

Second is that my parents were scholars in the humanities and they are themselves researchers. But their research is to read these old manuscripts and ask philosophical questions or questions related to literature. But because their students in the humanities had so much trouble finding jobs, I remember very vividly my parents saying, “Don't do a career in humanities because there are just no jobs. Science is the way to go.”

In France, you choose quite early what kind of broad field you want to follow. I was always oriented to more scientifically oriented courses, which I loved. I went to a sort of preparatory school and the courses were excellent.

And here was really the revelation for me about biology. I remember having discussions with other students who were more math and physics oriented. And I was talking about how the planet came about and how rocks were formed and transformed and how life was formed and transformed. And I was completely enthusiastic. And my friends doing physics were looking at me and saying, “Hm, I wish I could speak about physics with that much enthusiasm.”

**Gitschier:** Where did you do your PhD?

**Dulac:** I did it in a developmental biology lab, and the question was very basic: how do cells decide their fate? And it was an absolutely terrific experience, both because the project was fascinating, but also because my mentor was a really exceptional person. Nicole Le Douarin is one of the most famous developmental biologists, not only in Europe, but also in the world. I learned enormously from her—not only about science, but thinking about science, and teaching as well. She was extremely enthusiastic about science, and I had this in common with her. She was also a terrific role model.

After my PhD, the big question was, what do I do next? And for me, there were a number of very pressing considerations. One is, the work I was doing was in non-molecular, non-genetic model systems, which are the quail and the chicken embryos. But I'm a very mechanistic person; I wanted to go into a model system that is very molecular or very genetic, and that is obviously the mouse.

And the other consideration… My parents in their infinite wisdom had decided that I wouldn't need to learn English in school because I would have to learn it someday anyway. So I therefore took Latin and Ancient Greek and German and Russian as my four languages. But that was terribly handicapping because I needed to read and speak English and I had no clue. I bought some tapes and it was just excruciating. The first time I got off the plane in the United States I had never spoken a single word of English—*ever*!

So, this is just to say something that might seem very obvious here, but was not obvious in France at the time, which is that I needed to do a post-doc after my PhD, and that I needed to do it either in England or in the US. And the heart of research is in the US.

And that's how I ended up doing a post-doc in Richard Axel's lab at Columbia.

**Gitschier:** Well, there are many places in the US to work on mice. What were the other pieces of the puzzle?

**Dulac:** I visited many labs in the US. What attracted me a lot to Richard Axel's lab was that when I was looking, in 1992, Richard and Linda [Buck] had just published the cloning of the olfactory receptors. But what really did it was the extraordinary personality of Richard Axel. Someone who is extraordinarily thoughtful and likes to ask big questions, with this analytical and creative mindset to phrase questions in a way that they now become addressable. And the field of olfaction was stuck, because people were trying to address the most complex questions right away. It was a complete mess.

**Gitschier:** Was it Richard's idea to go after the olfactory receptor, or Linda Buck's idea?

**Dulac:** I don't exactly know what the history is, but my understanding is that Linda was one out of many post-docs who had tried to clone these receptors in Richard's Lab. But the methodology used to get at them—she's the one who set up this very clever PCR approach for conserved regions of seven-transmembrane receptors.

**Gitschier:** Had you yourself heard of the VNO before?

**Dulac:** Richard was the one talking about it. Richard is very funny and politically incorrect. He had this dream of cloning the mating receptors—the VNO receptors—and exchanging them with the receptor for lemon. And then having a mouse mounting a lemon. And that, he said, would be the cover of *Cell*.

The serious idea behind it was to clone genes that were associated with innate behavior, and for him that was fascinating. And no, I had never heard of the VNO before!

What I found fascinating with the VNO was the development of these pre-programmed behaviors. Olfaction, smell—you learn to smell. But you don't *learn* pheromones.

**Gitschier:** I wouldn't even know how to detect a pheromone.

**Dulac:** You wouldn't detect them consciously. And that's the whole idea. The VNO completely bypasses conscious areas. It never hits the cortex. Nothing is processed through cognitive areas; it is completely innate. It goes to the amygdala and the hypothalamus, and *boom*—you either mate or attack. It is a subcortical pathway that controls a central behavior.

So the idea with pheromone receptors was that they would be easy [to clone], just a subclass of olfactory receptors.

**Gitschier:** And so did you try Linda's approach?

**Dulac:** When I came to Richard's lab, I had two projects: a difficult project and an easy project. Both of them were impossible.

The difficult one was to figure out how olfactory neurons choose to express one receptor. That problem has still not been solved—almost 20 years later.

Then I had the easy project that ended up being as impossible as the first one! And that was to identify the pheromone receptors. And everyone believed that would be an easy project, because everyone assumed that the pheromone receptors would just be a subfamily of the olfactory genes. And indeed, when Linda used the PCR primers she had designed and amplified stuff from the VNO, where the pheromone receptors are expressed, she was able to get receptors out of it.

But there was something just wrong. Because when I started to look at the expression of these genes in the VNO, less than 1% of the neurons were expressing olfactory receptors, so there had to be something else.

So here is how my 2 or 3 years of failure came about because there was “no doubt” that the pheromone receptors had to be something related to the olfactory receptor, maybe a bit distant family. But I tried the Linda Buck approach again and again and again. I thought, “I'm a failure.”

**Gitschier:** And Linda had left the lab at this point?

**Dulac:** Linda had left the lab and much later, I got to know a person, Emily Liman, a post-doc in Linda's lab, who was trying to do exactly the same thing at the same time, and also failed for years. We actually ended up many years later collaborating very closely on identifying essential players of VNO signaling.

And I thought, maybe I'm a failure, maybe I'm incompetent—but not *that* incompetent. So there has to be something else.

**Gitschier:** You must have been pretty down in the dumps at that point.

**Dulac:** Yeah, I had times that were really hard. Well, here is where Richard is quite extraordinary. Richard would always say, “I don't know when you will be able to make a hit, but I know you will.”

**Gitschier:** Wow!

**Dulac:** “I don't know if it's going to be tomorrow, I don't know if it's going to be in 5 years.”

**Gitschier:** What made him know that?

**Dulac:** Well, you know, Linda cloned the olfactory receptors after 10 years as a post-doc. So that totally relieved the time pressure.

**Gitschier:** So he was saying that he would support you no matter what.

**Dulac:** Yes. What was important was to work on a big project, an important project, and he would be supporting you as long as you go for it.

And so at some point I did a key experiment, I think, which *a posteriori* is so simple. Now, Linda was able to clone the olfactory receptor genes because she knew what was the signal transduction. This was published: activation of olfactory neurons would lead to a cyclic nucleotide pathway and from this you would have a cyclic nucleotide-gated channel, and that would be the beginning of the electrical, neuronal signal. And so, because she knew this, she could assume the receptors were GPCRs [G-protein-coupled receptors], and therefore she designed primers based on the known GPCRs.

So that was also our assumption for the VNO, but because there was just no way I could get these god-damn things, I said, “Maybe the signal translation is just not cyclic nucleotide-related.”

And so I did a very simple experiment. I had a cDNA library and I just looked for the signal transduction molecules that were known in the olfactory system. I had an olfactory library and I had a VNO cDNA library, and if you hybridize filters with a cyclic-nucleotide gated channel probe, in the olfactory system about 1% of the phages are clones for the channels, but if you do the same experiment in the VNO, there is nothing, absolutely nothing.

**Gitschier:** So you basically backed up.

**Dulac:** I backed up and I re-asked the question. Are our assumptions about the nature of this receptor accurate? And they were not. None of the key elements of the olfactory signaling pathway—none of them—were there.

So I didn't know where these pheromone receptors were; I didn't even know what family they belonged to. They could be receptor tyrosine kinases or phosphatases. So I had to come up with a strategy that made no assumption.

There were two possible strategies. In differential display, you would sequence everything. I forget how it worked but there was something extraordinarily labor-intensive.

Or there was a very elegant method that seemed totally nuts, crazy—that was to make libraries from a single neurons. And that was based on the idea that in the olfactory system, every neuron expresses a different olfactory receptor gene, and the expression at the cellular level is very high, but since every cell expresses a different receptor, the organ is a mosaic. So if that were the case for the VNO as well, then by comparing either two VNO neurons or a VNO and an MOE neuron, I would get the VNO receptor.

So I went to Richard's office one day and I said, “I know how I'm going to get them.”

And he said, “Well, you told me that already, several times.”

So this was another idea. He said, “OK, what is the idea?”

And I said, “I'm going to make single-cell libraries and by differential screening, I'm going to get the receptors.”

“OK, well come back when it works.” He thought the idea was extremely interesting, but it seemed totally impossible.

It was interesting because within the lab there was a lot of competition, but this strategy of single-cell libraries seemed to be so crazy that suddenly I was the nutcase in the lab. And it was great because people left me alone. There was no competition any more, because I was doing this completely unfeasible thing. And that was fine with me. I could just think calmly about stuff and go through the steps of making the library.

And one day, I found some clones that were differentially expressed. And I took the clone and I did an *in situ* hybridization with a VNO section, and they had exactly the right pattern. Ah! But the signal was weak because the clone was very small, and I was not willing to show that to Richard, because I knew he would look at it and say “Puh.”

So I waited to get a longer clone and do another *in situ* that would look better. I kept my finding a secret for a month or 2 months, which was bizarre because I'm not a secretive person. On the other hand, there was some real satisfaction to be the only one in the world to know that you got it!

Then, that was an interesting time of my life because I had no doubt that I would do a post-doc in the US and then come back to France.

I love France. The idea of staying in a foreign country was impossible to me. However, there was a problem. I could see from being at Columbia what people after doing a successful post-doc would get: an independent lab, an independent budget, etc. And when I tried to see what I could get in France, it was very clear that all I could get was to go into somebody else's lab with maybe a bench or so. And it made it impossible for me to come back to France right away.

I feel very grateful to my country that gave me what I consider to be an incredibly high quality education. And I feel that we serve our countries, and for me going back to France and being a scientist and teacher in France was my way of giving back. But somehow France wasn't offering me the chance to do so.

It is tough when you go through a PhD and a post-doc with absolutely top science that you are then being asked to forget all of this because you are not given the means to achieve the top level in France.

**Gitschier:** Are you a US citizen now?

**Dulac:** Not yet. I should be, and I promised that if Obama got elected I would become a US citizen. So I have some work to do!

**Gitschier:** Somehow your interests have now led you to questions of genetic imprinting. I have to tell you, I was so impressed that you wrote a review on epigenetics because…

**Dulac:** …it had nothing to do with my work.

**Gitschier:** Right! Let's talk about your move into that area now.

**Dulac:** My interest in the VNO and behavior is not only male versus female, although there is that, but it is more this idea of an animal being born and being able to recognize who are the members of their own species, who they are supposed to mate with, who they are supposed to attack, who they are supposed to nurse, who is a predator. These so-called innate behaviors.

The animal needs to know what to do and that information in a large part comes from the genome. If you're a mouse and you need to *learn* what is a predator, then you are dead, because you don't have a chance to learn.

So there must be a certain amount of information, provided by your genome, on how to respond to certain aspects of the environment, and this complex interaction between the genome and brain function is something that I find fascinating. And it has been fascinating me for a very, very long time.

**Gitschier:** Before you went to Richard's lab.

**Dulac:** Yes. Even as a kid. The pheromone system for me was a big discovery because I ran into a question that was very dear to me, and I could even work on it for my profession!

And males and females respond to stimuli in completely different ways, and in most animal species, the difference in behavior is *dramatic*. But if you look at the brain, the male and female brains look alike.

However, Tali Kimchi in my lab showed that if you remove the VNO of the female, she will start to behave like a male. Which says is that the brain is essentially the same, and what the VNO does is tell an animal to behave like a male or a female.

So the epigenetic part belongs to the same question on the relationship between genes and behavior. What epigenetic changes are for the brain are changes that enable different states of behavior. So for example, post-traumatic stress—it looks very much like a brain that is taking a different path in terms of functioning of behavior circuits and then is stuck in that path. You can see depression or mood disorders that way. Puberty is something similar. Puberty comes at the time when the brain circuitry is almost achieved; however, there is something happening that is making the brain of a young animal and the brain of a slightly older animal do completely different things!

I am intrigued with these changes of states of the brain. And epigenetic changes are something that goes on top of the genomic information and enables certain genes to change their expression in a very stable way. The phenomenon of genomic imprinting seemed particularly interesting for this, as it is a known epigenetic regulation in placental mammals, in which early maternal or paternal alleles of certain genes are expressed.

Now, having two copies of each gene is a huge evolutionary advantage, because if anything goes wrong with one gene, another comes to the rescue. So the question is, why would animals choose to silence one copy of essential genes?

My colleague David Haig had proposed a model: what is very special about placental mammals is that moms do most of the job in providing all the resources in raising the embryo, as long as the embryo develops *in utero*. And that sets up a conflict between the maternal and paternal genomes, because dad is trying to favor the growth of its progeny by trying to suck as many resources from mom as possible, while mom needs resources for her and for future embryos. This hypothesis was proposed even before the first imprinted genes were discovered! And so 1 year later, the first two imprinted genes were discovered, and they were *Igf2*—paternally expressed—a growth factor that promotes embryonic growth, and *Igf2r*, its receptor, which is a truncated receptor that antagonizes embryonic growth and is maternally expressed.

I started to think about brain development and brain function, and in principle there is no reason that early imprinting would affect only embryonic growth. It should also affect the behavior of the newborn animal, and maybe even the adult. That's why Chris Gregg, a post-doc in my lab, and myself, in collaboration with David Haig, started to be interested in genomic imprinting in the brain and its impact on behavior. And the fact that we found that some imprinted genes are imprinted only in males or only in females was not something we expected, at all.

It is particularly interesting for brain function because most of the brain disorders have different prevalence in men and women. So depression, MS [multiple sclerosis], eating disorders, schizophrenia, you name it. The fact that you have genes that are present at one copy in one sex and two copies in the other sex would certainly offer a trove of candidates for genes being implicated in these diseases. So that was a great finding.

**Gitschier:** Well, what were you expecting?

**Dulac:** We first asked the question: is there anything special about imprinting in the brain and behavior. If our hypothesis was that imprinting could also have been selected evolutionarily to modulate certain behaviors, then imprinted genes must be expressed in certain specific brain areas. So there must be something special about the expression of imprinted genes compared with the expression of biallelic genes.

And what we did was to use the Paul Allen Brain Atlas that maps gene expression in the brain, and we looked at all the imprinted genes, and we took randomly selected biallelic genes.

**Gitschier:** Known previously.

**Dulac:** Yes—absolutely. If there is something special about genomic imprinting and behavior, then imprinted genes should be preferentially expressed in certain brain areas compared to normal biallelic genes.

We took genes one by one, went through all the sections, and looked at whether gene 1 was expressed in brain area a, b, c, d, and we did 120 brain areas. A fantastic undergraduate student actually did all this work, Brady Weissbourd, together with Chris Gregg.

And something very striking emerged from those studies—the majority of the biallelic genes are expressed in the cortex. In other words, the cortex is the place in the brain where there is the maximal molecular complexity. But if you look at imprinted genes, the hot-spots are the hypothalamus, the amydala, the dorsal raphe, all the areas that are involved in pain sensation, eating behavior, social behavior, something literally completely non-overlapping with the biallelic genes.

**Gitschier:** So once you had analyzed the data, it was even more impressive than you imagined.

**Dulac:** Yes, but this was an *in silico* experiment. It didn't tell us whether there were other imprinted genes that had yet to be discovered in those brain areas, and so that was the next question. There was another really important aspect of the experiment—what brain areas to start with.

Here is a very cool experiment published in 1995 by Barry Keverne in England. What he and his collaborators did was to take zygotes with duplication of maternal or duplication of paternal genome and to mingle them with normal embryonic cells—genetic chimeras. These give rise to full-blown embryos and are born. Then you can look at the brains of these chimeras, and the results are absolutely fascinating.

Have you ever heard of these experiments?

**Gitschier:** Not these that you are describing now.

**Dulac:** So here's what happened. With the entire paternal duplication embryos have large bodies and small brains. The embryos with duplication of the maternal genome have small bodies and large brains.

Then you can look at different parts of the brain, ask what brain areas the maternal and paternal genomes contribute to preferentially, and that result is absolutely fascinating: cells with duplication of the paternal genome go exclusively to the hypothalamus and avoid the cortex, but cells with duplication of the maternal genome go exclusively to the cortex and avoid the hypothalamus. What this says is that the paternal genome is required for the hypothalamus and a maternal genome is required for the cortex.

Barry Keverne's hypothesis was that dads only think about sex, and that's what the hypothalamus does, and moms think about social interaction, maternal care, which requires the cortex.

When I explain this experiment, guys say, “Oh my God”—and women love it! But what that told us is that if we were to be looking for imprinted genes in the developing brain, in the adult cortex and the adult hypothalamus, the prediction was that we would find more maternally expressed genes in the cortex and more paternally expressed genes in the hypothalamus.

**Gitschier:** It's really interesting. Now I just wanted to ask you something that is always in the news, even though we're not talking about male versus female brains…

**Dulac:** Well, so one aspect of our story is that some of these are imprinted only in males or only in females.

**Gitschier:** Right. So I was going to ask you about Larry Summers.

**Dulac:** So we actually call the Keverne experiment of the maternal contribution to the cortex the “Larry Summers experiment.” In fact, I was reading the Keverne paper at exactly the same time as Larry Summers was making his comments about women not being able to do science.

And in fact, some of my male students remember the experiment the other way around, that there is a preferential paternal contribution to the cortex. There is a total disconnect between their expectation and the result. Fascinating!

